# Cell Death and Inflammation: The Role of Mitochondria in Health and Disease

**DOI:** 10.3390/cells10030537

**Published:** 2021-03-03

**Authors:** Anna Picca, Riccardo Calvani, Hélio José Coelho-Junior, Emanuele Marzetti

**Affiliations:** 1 Fondazione Policlinico Universitario “Agostino Gemelli” IRCCS, 00168 Rome, Italy; anna.picca@guest.policlinicogemelli.it (A.P.); emanuele.marzetti@policlinicogemelli.it (E.M.); 2 Aging Research Center, Department of Neurobiology, Care Sciences and Society, Karolinska Institutet and Stockholm University, 17165 Stockholm, Sweden; 3 Università Cattolica del Sacro Cuore, Institute of Internal Medicine and Geriatrics, 00168 Rome, Italy; coelhojunior@hotmail.com.br

**Keywords:** apoptosis, damage-associated molecular patterns (DAMPs), immunogenic cell death, innate immunity, mitochondrial dysfunction, mitophagy, mitochondrial dynamics, mitochondrial quality control (MQC), oxidative stress, reactive oxygen species (ROS)

## Abstract

Mitochondria serve as a hub for a multitude of vital cellular processes. To ensure an efficient deployment of mitochondrial tasks, organelle homeostasis needs to be preserved. Mitochondrial quality control (MQC) mechanisms (i.e., mitochondrial dynamics, biogenesis, proteostasis, and autophagy) are in place to safeguard organelle integrity and functionality. Defective MQC has been reported in several conditions characterized by chronic low-grade inflammation. In this context, the displacement of mitochondrial components, including mitochondrial DNA (mtDNA), into the extracellular compartment is a possible factor eliciting an innate immune response. The presence of bacterial-like CpG islands in mtDNA makes this molecule recognized as a damaged-associated molecular pattern by the innate immune system. Following cell death-triggering stressors, mtDNA can be released from the cell and ignite inflammation via several pathways. Crosstalk between autophagy and apoptosis has emerged as a pivotal factor for the regulation of mtDNA release, cell’s fate, and inflammation. The repression of mtDNA-mediated interferon production, a powerful driver of immunological cell death, is also regulated by autophagy–apoptosis crosstalk. Interferon production during mtDNA-mediated inflammation may be exploited for the elimination of dying cells and their conversion into elements driving anti-tumor immunity.

## 1. Introduction

Mitochondria are multitasking organelles involved in energy supply, cell death/survival signaling, iron and calcium buffering, reactive oxygen species (ROS) signaling, and steroid hormone and heme biosynthesis. To ensure that all these tasks are efficiently accomplished, organelle homeostasis needs to be ensured at all times. To this aim, a set of mitochondrial quality control (MQC) processes, including organelle’s dynamics, biogenesis, proteostasis, and autophagy, is in place to safeguard mitochondrial integrity and functionality [1].

Failing MQC has been documented in several age-related conditions, and mitochondrial dysfunction has been indicated as a relevant mechanism in chronic low-grade inflammation associated with aging (i.e., inflammaging) [2,3]. In particular, fragmentation and release of mitochondrial DNA (mtDNA) have been proposed as a trait d’union between mitochondrial dyshomeostasis and inflammaging [4,5,6]. Indeed, aberrant CpG methylation motifs within mtDNA make these molecules recognized as “non-self” and, thus, as inflammatory triggers [5,6]. Displaced mtDNA and mitochondrial nucleoids have been detected in the setting of numerous disease conditions [7,8]. However, the mechanisms regulating their unloading are still insufficiently understood. The most accredited hypothesis posits that a danger signaling response ignited by a stressor harnesses damaged mtDNA as an alert molecule to instigate a pro-homeostatic signaling cascade [9,10].

Among other stressors, a burst of mitochondrial ROS and ensuing oxidative stress can elicit inflammation via the activation of nuclear factor κB (NF-κB) [11]. ROS overproduction may also trigger the opening of the mitochondrial permeability transition pore (mPTP), impinge on mitochondrial dynamics and mitophagy, and induce programmed cell death [12]. Upon mPTP opening, dysregulated MQC processes may result in extracellular delivery of mtDNA [13,14].

The leakage of mitochondrial components (e.g., mtDNA, cardiolipin) into the cytosol and outside the cell may act as damage-associated molecular patterns (DAMPs) [15] and an innate inflammatory response can be mounted [9]. Indeed, the exposure to these cellular debris allows recruitment of adaptor molecules/receptors that triggers an innate immunity response [9]. In this regard, the recognition of DAMPs by cyclic GMP–AMP (cGAMP) synthetase (cGAS), toll-like receptor 9 (TLR9), and the NLRP3 inflammasome is pivotal for eliciting inflammation [16].

According to a recently proposed mitochondrial theory of aging [17], cytosolic mtDNA fragments can be translocated into the nucleus. Here, mtDNA can be incorporated within nuclear DNA and contribute to genomic instability [18,19]. Insertions of mtDNA at the level of the pericentromeric area of chromosomes have been observed during aging in yeasts and rodents [18,19]. An increase abundance of pericentromeric mtDNA inserts has been indicated as a contributor to accelerated aging [18,19]. The nuclear localization of mtDNA fragments may also affect nearby centromeres and induce chromosome disaggregation during mitosis [20,21]. Moreover, mtDNA inserts can migrate from the pericentromere where they are more abundant to other chromosomal regions and potentially cause cancer and other diseases by altering nuclear DNA sequence [20,21]. Remarkably, the administration of rapamycin, a compound that extends rodent longevity, reverses the age-related accrual of mtDNA in the nucleus and the deposition of the age pigment lipofuscin in the cytoplasm [22].

Here, we overview the potential routes of mtDNA displacement that have been described so far and discuss their involvement in physiological and pathological conditions. A special focus is placed on the emerging role of mtDNA unloading in priming anti-tumor immunity via immunological cell death.

## 2. Altered Mitochondrial Quality Control Pathways Are Routes of Mitochondrial DNA Displacement

MQC processes integrate organelle’s dynamics, biogenesis, proteostasis, and autophagy that collectively assist in preserving metabolic cellular “fitness” and plasticity [23]. In particular, coordinated cycles of fusion and fission support mitochondrial plasticity to match cellular energy demands and the buffering of mtDNA damage [24]. These activities, in coordination with autophagy, dispose bioenergetically defective organelles with hyper-fissioned mitochondria being segregated from the network and tagged for degradation within lysosomes [24]. Concomitantly, mitochondrial replenishment via organelle biogenesis ensures the maintenance of an efficient mitochondrial pool within the cell [24].

As an additional level of mitochondrial quality check, the establishment of tubular protrusions (i.e., mitochondrial nanotunnels) and the generation of mitochondrial-derived vesicles (MDVs) have recently been described [25,26]. Mitochondrial nanotunnels allow mitochondrial interconnections and inter-mitochondrial content exchange over long distances [25,27]. These structures are especially relevant in post-mitotic tissues (e.g., skeletal muscle, myocardium) in which mitochondria are immobilized and fusion is limited [28]. MDV generation, instead, has been indicated as a housekeeping mechanism that complements mitophagy and recycles damaged, but not yet depolarized organelles along the endocytic pathway [27].

Of note, altered mitophagy and dynamics have been listed among the most accredited routes implicated in mDNA unloading and are discussed in the following sections.

### 2.1. Mitochondrial Dynamics

The coordination of fusion and fission events supports mitochondrial dynamics to regulate organellar shape, metabolic plasticity, redox homeostasis, and cell death/survival [29,30]. Mitochondrial fusion enables organelle networking, the mixing of mtDNA and metabolites, and bioenergetic inter-organelle signaling [31]. Via this process, mutant/oxidized mtDNA molecules are diluted along the network and bioenergetic deficits may be complemented [31]. Mitochondrial fission ensures equal organelle segregation between daughter cells and singles out bioenergetically inefficient mitochondria to be targeted for mitophagic removal [24]. Concomitant with altered dynamics, abnormally shaped and defective mitochondria have been identified under stressful conditions and in cells acquiring a senescent phenotype [32]. Elongated mitochondria producing high levels of oxidants and expressing low levels of fission protein 1 (FIS1) have been observed in cultured cells treated with hydrogen peroxide [32]. Impaired mitochondrial function has also been documented in hyper-fissioned, "dot-like" mitochondria [33].

Dysmorphic, hyper-fused or hyper-fragmented mitochondrial phenotypes have been observed in several diseases characterized by inflammation and mtDNA release [5,6,14,34,35]. Indeed, impaired mitochondrial dynamics may cause mtDNA instability via the formation of altered nucleoid structures as a result of abnormal packaging. These aberrant conformational changes can trigger inflammation via mtDNA unloading [36]. Giant mtDNA nucleoids have been observed in hyper-fissioned mitochondria of models of optic atrophy 1 (Opa1) ablation with TLR9-driven inflammatory response [37]. Conversely, the appearance of hyperfused elongated mitochondria and reduced apoptosis have been documented in cells with dynamin-related protein 1 (Drp1) depletion [38]. The ablation of the mitochondrial transcriptional factor A (Tfam), a component of mtDNA nucleoids, induces the formation of hyperfused mitochondria that are prone to releasing mtDNA fragments into the cytosol [39]. These fragments frequently encompass the non-coding D-loop region of mtDNA [39]. Altered TFAM binding to this mtDNA region has been indicated as a possible mechanism underlying mtDNA dyshomeostasis and as an additional route of mtDNA release [40]. Although the exact mechanisms through which mtDNA is displaced following altered dynamics are unknown, the release of mtDNA fragments from oxidatively stressed organelles seems to occur via pores formed by voltage-dependent anion channel (VDAC) oligomers [41].

The coordination between fusion and fission, besides shaping the mitochondrial network, is a checkpoint for cell’s death/survival [42]. In particular, the depletion of Opa1 triggers apoptosis in cultured cells [42], while Fis1 or Drp1 silencing represses mitochondrial fragmentation and the execution of programmed cell death [42]. Aberrant mitochondria are often found in aged tissues indicating a dysregulation of mitochondrial dynamics in advanced age. These age-related morphological abnormalities are accompanied by changes in the expression of mitofusin (Mfn) 1 and 2, Opa1, Drp1, and Fis1 [43,44,45,46]. Mutations of Opa1 and Mfn2 are also causative factors of the two neurodegenerative disorders, dominant optic atrophy (DOA) [47] and Charcot-Marie-Tooth type 2A (CMT2A) [48]. Mice harboring Opa1 mutations show neuromuscular defects related to axonal and myelin degeneration resembling those found in people with DOA [49]. A role for MFN signaling in muscle homeostasis has also been suggested. In particular, protein levels of MFN2 was found to be reduced in human muscles under several catabolic conditions [33,45,50].

A shift of mitochondrial dynamics towards fission has been observed in several conditions in humans, including hip fracture-associated muscle atrophy and muscle wasting during gastric cancer-related cachexia [45,51,52]. Conversely, the promotion of fusion in very old rodents maintains mtDNA homeostasis and possibly serves as a pro-longevity mechanism [53]. A systemic inflammatory response syndrome (SIRS), characterized by increased circulating levels of cell-free mtDNA, has been documented in patients with hip fracture [54]. In this context, circulating mtDNA may promote the development of inflammation by recruiting leucocytes [54].

### 2.2. Mitophagy

Mitophagy is a degradative process that starts with the engulfment of a mitochondrion into a double-membrane structure called the autophagosome and culminating in organelle degradation after fusion with lysosomes [55].

Serin/threonine-protein phosphatase and tensin homolog-induced kinase 1 (PINK1)/Parkin-dependent and independent pathways are involved in the regulation of mitophagy [56]. However, PINK1/Parkin-dependent mitophagy is the best characterized degradation pathway in mammalian cells. In healthy, well-functioning mitochondria, PINK1 is taken up via the translocase of the outer mitochondrial membrane (TOM) and the translocase of the inner mitochondrial membrane 23 (TIM23), and is subsequently cleaved by the presenilin-associated rhomboid-like (PARL) protease [57,58,59]. Conversely, in uncoupled mitochondria, PINK1 accumulates at the outer mitochondrial membrane (OMM) where it is stabilized [58,60]. Here, the autophosphorylation of PINK1 at specific serine residues enables its activation [32] and guides the recruitment of Parkin from the cytoplasm to the OMM [57,61,62,63,64,65]. Upon its localization at the OMM, Parkin ubiquitinates several proteins including VDAC, Ras homolog family member T1 (RHOT1), and MFN1 and 2 [57]. The polyubiquitination of these substrates enables their interaction with mitophagy adapters (i.e., nuclear dot protein 52 (NDP52) and optineurin (OPTN)) and the microtubule-associated protein 1A/1B-light chain 3 (LC3) [66,67]. This step is mediated by the recognition of WXXL motifs that prompt mitochondrial delivery to autophagosomes upon organelle sequestration within an autophagosomal membrane [66,67,68]. This process is mediated by the accumulation of the ubiquitin-binding adaptor protein p62/sequestosome-1 on mitochondria targeted for disposal and its binding to LC3 [68]. As a final step, the delivery of mitochondria to autophagosomes occurs and their content is degraded upon fusion with lysosomes [68]. Through this pathway are also processed mitochondria containing damaged mtDNA which is degraded within autolysosomes by DNase II [69].

Altered mitophagy has been associated with either damaged mtDNA accrual within mitochondria or its release towards the cytosol or the extracellular compartment. Of note, in these as well as in any other circumstances overwhelming the autophagic machinery, inflammation may be triggered [70]. The regulation of the expression of the intracellular danger sensor TLR9 may be the missing link in this response. Indeed, TLR9 is synthesized within the endoplasmic reticulum (ER) and is directed towards the endo-lysosome for DNA recognition [70]. Here, the interaction between TLR9 and digested DNA induces a type I interferon (IFN-I)- or NF-κB-mediated inflammatory response [70]. The accumulation of mtDNA into autolysosomes and the co-localization and activation of TLR9 have been observed in DNase II-deficient mice, a model of heart failure, suggesting a relationship between TLR9-induced inflammatory response and disease pathogenesis [14]. Inflammation has also been observed independent of mtDNA relocation within the cytosol or the extracellular space, which suggests that the release of mtDNA within these compartments be not mandatory to trigger inflammation [14]. In this scenario, crosstalk between mitophagy and the endo-lysosomal system may be crucial. Indeed, this checkpoint enables undigested mtDNA produced during DNase II deletion to interact with TLR9 and trigger inflammation [14]. Notably, an escape route for mtDNA from autophagosome recognition and DNase II-mediated degradation has also been reported in a mouse model of atherosclerosis [71].

The existence of an alternative system that operates independent of mitochondrial depolarization, autophagy, or mitochondrial fission and that signals via MDV generation and release, has been implicated in the disposal of mildly damaged mitochondria [72]. Indeed, MDVs can still be generated by cells lacking the autophagy-related serine/threonine kinase gene (Atg) 5, Beclin-1 or Ras-related in Brain protein 9 (Rab9) or with Drp1 silencing [72]. MDVs are small extracellular vesicles (EVs) of ~100 nm in diameter [73] budding from the endo-lysosomal system for the degradation of organellar components [72] and require the priming of PINK1 and Parkin for their generation [73]. As such, MDVs cooperate with mitophagy for MQC deployment when "canonical" degradative routes are overwhelmed or compromised [74]. Although the molecular events leading to MDV generation are largely unknown, the most accredited hypothesis involves the deposition of oxidized mitochondrial components in close proximity of the organelle’s membranes [73]. Under oxidative stress conditions, this relocation together with cardiolipin oxidation may induce conformational changes of mitochondrial membranes, most likely unconventional curvatures, that interfere with the correct assembly of import channels at these sites [73]. These abnormal membrane curvatures may induce the accumulation of PINK1 at the OMM with the consequent recruitment and ubiquitination of Parkin [73]. The finalization of vesicle formation and release is mediated by a set of yet unidentified proteins [73]. Cells harboring only mild mitochondrial defects shuttle functional mitochondria within MDVs to rescue aerobic respiration, thereby indicating a link between mitochondrial defects and EV endocytosis [75,76,77,78]. Mitochondrial constituents have also been found to be displaced within MDVs in age-related conditions characterized by altered cell’s quality control mechanisms [79,80,81]. MDV generation, orchestrated by mitochondrial–lysosomal crosstalk, has also been recognized as a candidate mechanism linking cellular dyshomeostasis with systemic inflammation in the context of aging and associated conditions [2,80,81]. Indeed, in the setting of altered MQC processes, a defective clearance of dysfunctional organelles may lead to the leakage of noxious material via MDVs, which ultimately triggers inflammation [2]. Indeed, released molecules can be recognized and bound by membrane or cytoplasmic molecules that are referred to as pattern recognition receptors (PRRs) and act as immune sentinels. Following this binding, PRRs become activated and induce the release of INFs, pro-inflammatory cytokines, and chemokines as part of an innate immune response. However, whether displaced mtDNA operates via these routes and whether this pathway is involved in the pathogenesis of age-related conditions warrant further investigation.

**Figure 1:**
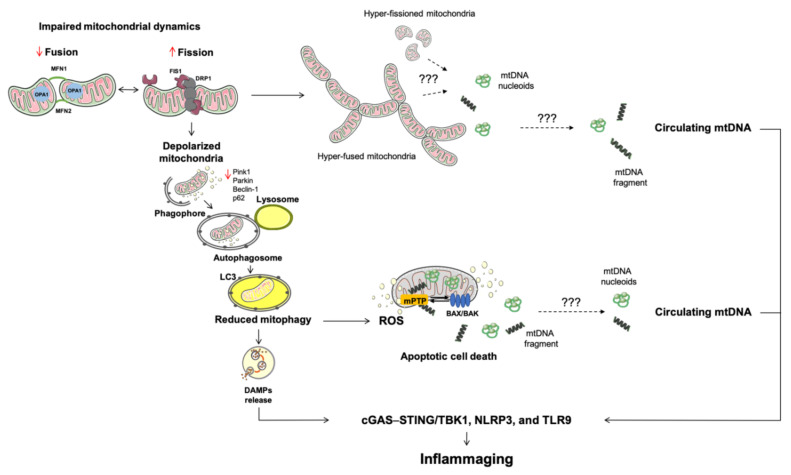
Schematic representation of altered mitochondrial quality control processes during aging and associated conditions. A multi-pathway derangement involving mitochondrial dynamics, mitophagy, and apoptosis characterizes mitochondrial dysfunction during aging and leads to cellular stress. Under these circumstances, the release of DAMPs ignite inflammation via innate immunity. Among other DAMPs, mtDNA is a strong pro-inflammatory molecule recognized by pattern recognition receptors. Of all mechanisms studied, the activation of BAK and BAX and the opening of the mitochondrial permeability transition pore are the best characterized route for the release of different molecules from mitochondria. Abbreviations: BAK, Bcl2 homologous antagonist/killer; BAX, BCL-associated X; cGAS–STING, GMP/AMP synthase—stimulator of interferon genes DNA-sensing system; DAMPs, damage-associated molecular patterns; DRP1, dynamin related protein 1; FIS1, mitochondrial fission 1 protein; MFN, mitofusin; mPTP, mitochondrial permeability transition pore; mtDNA, mitochondrial DNA; OPA1, optic atrophy protein 1; NLRP3, nucleotide-binding oligomerization domain-like receptor family pyrin domain containing 3; p62, sequestosome-1; PINK1, phosphatase and tensin homolog-induced kinase 1; ROS, reactive oxygen species; STING, stimulator of interferon genes protein; TBK1, TRAF family member-associated nuclear factor κB activator-binding kinase 1; TLR9, toll-like receptor 9.

Finally, mitophagy can be induced by ROS overproduction. In particular, ROS burst can trigger DNase II-driven degradation of oxidized mtDNA within autolysosomes. Oxidative damage can also saturate mitophagy and cause the release of oxidized mtDNA that may elicit inflammation via the endo-lysosomal TRL9 [82] or nucleotide-binding oligomerization domain (NOD)-like receptor family pyrin domain containing 3 (NLRP3) inflammasome signaling [83]. However, the mechanisms involved in mtDNA displacement and the consequent activation of inflammation in the setting of overwhelmed mitophagy are not fully elucidated. The opening of the mPTP and autophagic mitochondrial degradation may concur to displacing mitochondrial components [84,85]. Indeed, the release of damaged mtDNA via mPTP and its cytosolic translocation have been observed in the setting of mitophagy impairment [86]. The ablation of LC3B and Beclin-1, two mediators that regulate autophagosome initiation and maturation, causes altered autophagosome function and the accumulation of mtDNA under oxidative stress in macrophages [86]. Of note, the inhibition of mPTP with cyclosporin A and the treatment of macrophages with DNases mitigates mitochondrial dysfunction [86]. These findings indicate that mPTP may be a relevant mechanism for mtDNA release into the cytosol when mitophagy is altered [86]. In keeping with these results are those obtained in a mouse model of ventilator-induced lung injury in which mtDNA accumulation into autophagosomes, enhanced gene expression of mitophagy regulators, and higher circulating levels of cell-free mtDNA were associated with the activity of mPTP [8]. Therefore, at least in this context, the release of mtDNA into the circulation may represent the outcome of cell damage instigated by stressful conditions (Figure 1).

## 3. Apoptosis: A Converging Point for Cell Death and Survival

Under severe stress (e.g., growth factor withdrawal, extensive DNA damage, ER and replication stress, sustained ROS bursts, calcium overload, cytoskeleton alterations, mitotic defects), mitochondrial homeostasis is perturbed and the intrinsic pathway of apoptosis is triggered [87,88,89]. The initialization of this cell death program is under the control of pro-apoptotic proteins belonging to the B cell lymphoma (BCL) 2 family and containing the Bcl2 homology-3 (BH3) domain. Upon an apoptotic stimulus, BCL-associated X (BAX) and Bcl2 homologous antagonist/killer (BAK) are inserted into the OMM and oligomerize forming a membrane-spanning pore that allows efflux of apoptogenic factors [90,91]. The assembly of this pore structure induces profound conformational rearrangements in mitochondria that favor membrane permeability [91].

In the absence of stressors, BAX shuttles between the OMM and the cytosol in an inactive monomeric or dimeric conformation [92,93,94]. BAK is located at the OMM by inserting its C-terminal domain into the lipid bilayer and establishing a stable interaction with VDAC2 [95,96,97,98]. When apoptosis is triggered, the retrograde translocation of BAX to the cytosol ceases and the two pro-apoptotic proteins BAX and BAK become activated by pro-apoptotic BH3-only factors [99,100]. These include the BCL2 binding component 3 (also known as p53-upregulated modulator of apoptosis, PUMA), BCL2-interacting mediator of cell death (BIM), phorbol-12-myristate-13-acetate-induced protein 1 (also known as NOXA), and the BH3-interacting domain death agonist (BID). A transient interaction of these proteins with the mitochondrial pool of BAX and BAK leads to membrane conformational rearrangements that are required for pore formation [91].

Cells lacking BAX and BAK genes are resistant to a wide range of pro-apoptotic stimuli [101]. Furthermore, cells from BAX^−/−^BAK^−/−^ double-knockout mice do not activate the apoptotic program following growth factor withdrawal [102]. The survival of these cells is supported by autophagy through catabolism of intracellular substrates [102]. These findings indicate that, in case of growth factor or nutrient deprivation, cells activate autophagy to remain viable for a short period before programmed cell death is triggered. Indeed, OMM permeabilization initiates an irreversible cascade of events leading to mitochondrial translocation of BAX or BAK as an ultimate commitment to cell death [103].

A set of anti-apoptotic protein members of the BCL2 family including BCL2, BCL2 like 1 (BCL2L1 also known as BCL-XL), myeloid cell leukemia 1 (MCL1), BCL2 like 2 (BCL2L2, also known as BCL-W), and BCL2 related protein A1 (BCL2A1) is in place to antagonize OMM permeabilization [104,105]. These pro-survival factors possess all four BH domains and, similar to pro-apoptotic BAX and BAK, are inserted into the OMM or at the ER. Here, they serve anti-apoptotic functions by binding and blocking pro-apoptotic proteins of the BCL2 family [104,105]. Their pro-survival function is also related to their involvement in the regulation of ER calcium homeostasis [106], the promotion of bioenergetic metabolism via interaction with the F_1_F_O_ subunit of ATP synthase of the mitochondrial electron transport chain [107], and the modulation of redox homeostasis [108]. However, the majority of pro-survival BCL2 protein family members seem to inhibit BAX- and BAK-driven pore formation by their physical sequestration at the OMM or upon blockade of BH3-only activators [109].

In the setting of sustained OMM permeabilization, components of the mitochondrial membranes including cytochrome *C*, second mitochondria-derived activator of caspase (SMAC), and Omi (also known as high temperature requirement protein A2, HtrA2) are released, antagonize apoptosis inhibitors, and promote caspase-independent cell death. Indeed, the extrusion of cytochrome *C* leads to the organization of a structure called apoptosome that comprises cytochrome *C*, apoptotic protease-activating factor-1 (APAF-1), deoxyATP (dATP), and procaspase-9 [110,111]. Within the apoptosome, procaspase-9 is activated by oligomerization and subsequently engages apoptosis-executing caspases [110,111]. The release of cytochrome *C* and SMAC from mitochondria into the cytosol is mediated by cristae remodeling [112] via oligomerization and activation of the mitochondrial dynamin like GTPase OPA1 [112]. This molecular event seems to be preceded by BAX- and BAK-dependent activation of the zinc metallopeptidase OMA1 [113,114], and/or the fission protein DRP1 [112]. The nitrosylation of DRP1 has also been shown to precipitate the release of apoptogenic factors from mitochondria [115,116,117]. Once in the cytosol, cytochrome *C* binds to APAF-1 and procaspase 9 in a dATP-dependent manner to form the apoptosome [118]. Following caspase 9 activation, the proteolytic activation of the two executioner caspases 3 and 7 occurs. This event enables the enzymatic cell demolition via the intrinsic apoptotic pathway [119,120].

The inactivation of the X-linked inhibitor of apoptosis protein (XIAP) by the mitochondrial serine protease Omi/HtrA2 is an additional mechanism leading to apoptotic cell death. In particular, cytosolic SMAC binds to proteins of the inhibitor of apoptosis (IAP) family members, including XIAP [121,122,123]. The proteolytic cleavage and maturation of SMAC via the release of IAP-binding domain are required. This enzymatic activity is catalyzed by the inner membrane peptidase (IMP) complex [124] and the inner mitochondrial membrane PARL protease [125]. However, in the setting of mitochondrial dysfunction, cell death may also be triggered via OMM permeabilization when caspases are inactivated (i.e., caspase-independent cell death) to ensure cellular demise [103]. This pathway is discussed later in the article.

## 4. Mitochondrial Dysfunction in Innate Inflammation

An increase in IFN-I response during apoptosis has been observed in embryonic fibroblasts and hematopoietic stem cells from transgenic mouse models silenced for caspases 3 and 7, or 9 [126,127].

**Figure 2:**
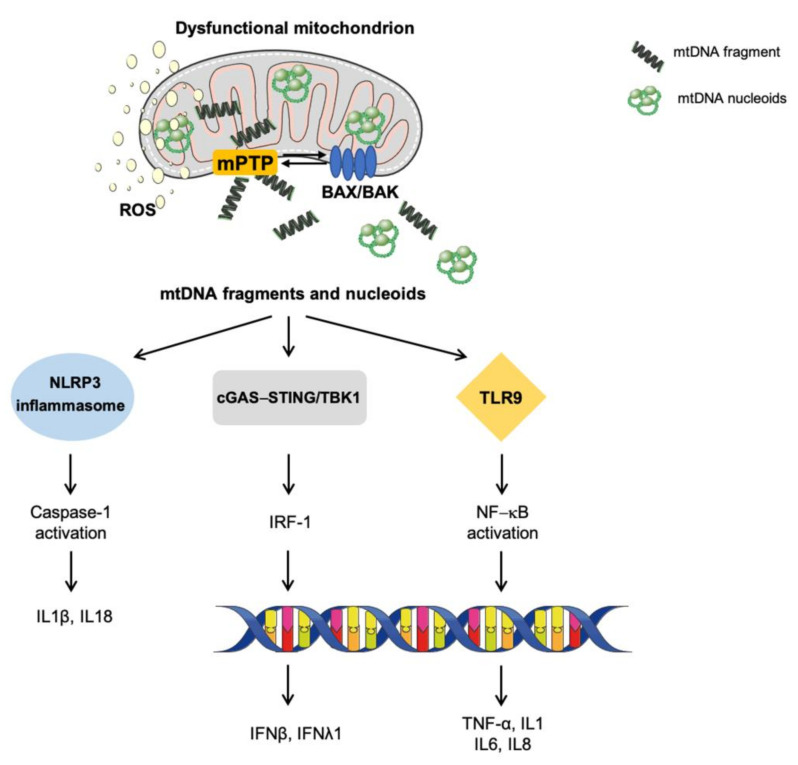
Signaling pathways eliciting sterile inflammation via damaged-associated molecular patterns. In the setting of mitochondrial dysfunction and cellular stressful conditions, the activation of BAK and BAX and the opening of the mitochondrial permeability transition pore enable the mitochondrial release of a set of molecules collectively named damaged-associated molecular patterns. Among these, fragmented and/or oxidized mitochondrial DNA bound to the mitochondrial transcription factor A (green circles) can trigger inflammation via the activation of three main pro-inflammatory routes: (1) toll-like receptors, (2) nucleotide-binding oligomerization domain-like receptor family pyrin domain containing 3 inflammasome, and (3) cytosolic cyclic GMP/AMP synthase—stimulator of interferon genes DNA-sensing system. Abbreviations: BAK, Bcl2 homologous antagonist/killer; BAX, BCL-associated X; cGAS–STING, GMP/AMP synthase—stimulator of interferon genes DNA-sensing system; IFN, interferon; IL, interleukin; IRF-1, interferon regulatory factor 1; mPTP, mitochondrial permeability transition pore; mtDNA, mitochondrial DNA; NLRP3, nucleotide-binding oligomerization domain-like receptor family pyrin domain containing 3; NF-κB, nuclear factor κB; ROS, reactive oxygen species; TLR9, toll-like receptor 9; TBK1, TRAF family member-associated NF-κB activator-binding kinase 1; TNF-α, tumor necrosis factor alpha.

This innate pleiotropic and adaptive inflammatory route is activated by mtDNA unloading and involves the activation of immunogenic cytosolic cyclic cGAS–stimulator of interferon genes (STING) pathway for the recognition of mitochondrial components. In particular, the binding of mtDNA to the DNA sensor cGAS generates the second messenger cGAMP that binds to the ER membrane adaptor STING [128]. This binding induces a conformational change of STING that becomes activated. In its active form, STING is translocated along the ER and the ER–Golgi network with the consequent recruitment and activation of TANK-binding kinase 1 (TBK1) by its carboxyl terminus. Once active, TBK1 phosphorylates the transcription factor IFN regulatory factor 3 (IRF3), thereby inducing its dimerization and translocation to the nucleus where it triggers a type I and III IFN response (β and λ1) and the transcription of IFN-stimulated nuclear genes [128] (Figure 2).

Besides cGAS–STING pathway activation, other immune sensing routes are elicited via binding to mitochondrial DAMPs. Indeed, mtDNA-induced inflammation has been reported to signal via TLRs and NLRP inflammasome activation. TLRs enable DAMPs binding to several antigen-presenting cells that become activated and trigger inflammation. In particular, TLR9 recognizes CpG domains within the mtDNA and leads to NF-κB translocation and an IFN-I response [14,129]. Of all NLRP classes, the best characterized NLRP3 is a cytosolic multi-component protein complex that guides caspase 1 activity and leads to interleukin (IL) 1β and 18 cleavage, with consequent engagement of macrophages, neutrophils, and T cells [130]. The activation of NLRP3 inflammasome by mtDNA has been observed during cell death. However, the synergistic action of inflammasome-mediated and redox-sensitive inflammatory pathways may reinforce the inflammatory response.

Finally, the nuclear translocation of NF-κB and the promotion of its transcriptional activity have also been included among the mechanisms eliciting a pro-inflammatory response [131]. Among the genes that are transcribed via this signaling pathway is tumor necrosis factor (TNF), a pro-inflammatory cytokine and an inducer of necroptosis, a regulated form of cell death sharing similarities with necrosis [131]. The activation of this pathway has been suggested to rely on BAX and BAK pores and VDAC oligomers, thereby implying an mPTP activation. However, the initiation of this response is unclear. Some lines of evidence indicate the existence of a mechanism operating via the release of the intermembrane second mitochondria-derived activator of caspase/direct inhibitor of apoptosis-binding protein with low pI (SMAC/DIABLO) through BAX/BAK. The extrusion of this pro-apoptotic SMAC protein binds and degrades IAP in the cytosol. This event triggers NF-κB signaling [131].

## 5. Immunological Cell Death: How Mitochondria Prime Anti-Tumor Immunity

Different from other forms of cell death (e.g., necroptosis and pyroptosis), caspase-dependent apoptosis is an immunologically "silent" process [132]. Indeed, the enzymatic activity of caspases involved in apoptosis, while ensuring the degradation of cell components, blocks the production and secretion of inflammatory cytokines by dying cells [133]. This, together with the deactivation of DAMPs signaling, avoids unnecessary immune activation of neighboring cells. With a similar goal, the engulfment of cellular component for lysosomal degradation is ensured by loss of cellular adhesion, cytoplasm fragmentation, membrane blebbing, and the exposure of pro-autophagy factors.

**Figure 3:**
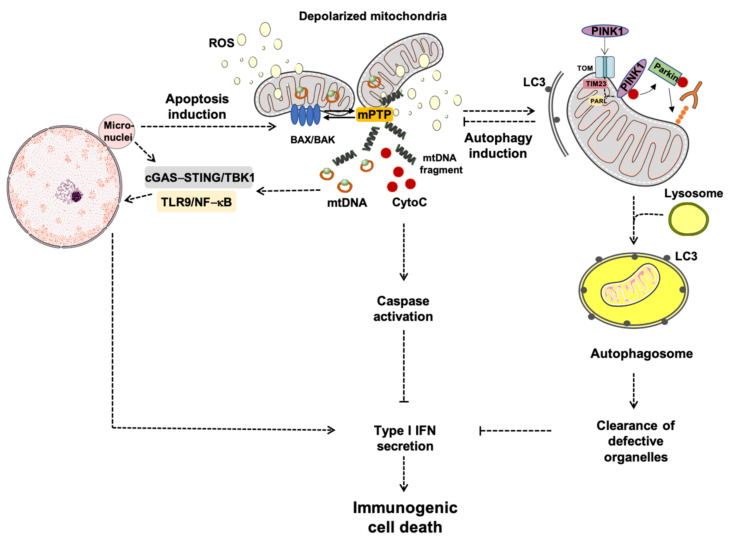
Schematic representation of apoptosis-driven caspase and autophagy activity eliciting immunogenic cell-death. Abbreviations: BAK, Bcl2 homologous antagonist/killer; BAX, BCL-associated X; cGAS–STING, GMP/AMP synthase—stimulator of interferon genes DNA-sensing system; CytoC, cytochrome *C*; IFN, interferon; LC3, microtubule-associated protein 1A/1B-light chain 3; mPTP, mitochondrial permeability transition pore; mtDNA, mitochondrial DNA; NF-κB, nuclear factor κB; PARL, presenilin-associated rhomboid-like protease; PINK1, phosphatase and tensin homolog-induced kinase 1; ROS, reactive oxygen species; TIM23, translocase of inner mitochondrial membrane 23; TLR9, toll-like receptor 9; TBK1, TRAF family member-associated NF-κB activator-binding kinase 1; TOM, translocase of the outer mitochondrial membrane.

As mentioned earlier, apoptosis is initiated upon activation of the two BCL2 proteins, BAK and BAX, and OMM permeabilization, through which mitochondrial components are extruded. These processes imply an extensive remodeling of mitochondrial membranes that favor organelle herniation to induce the exposure of matrix components, including mtDNA, to the cytosol [13,119]. These events translate into mtDNA-driven IFN production [134]. However, OMM permeabilization can also trigger autophagy to clear damaged cells along the endo-lysosomal pathway, which attenuates mtDNA-driven IFN production [134]. The caspase-dependent release of mtDNA or the inhibition of autophagy may convert this immunologically silent form of cell death into the so-called immunogenic cell death (ICD). Recent findings showed that mammary carcinoma cells from mice with genetic or pharmacologic autophagy inhibition had enhanced IFN-mediated inflammation in response to radiation therapy (RT) [135]. The results differ from those previously reported in which the pro-inflammatory response following RT was mainly attributed to the generation of micronuclei via DNA damage and genome instability [136,137]. However, confocal microscopy experiments showed that, following RT, cytosolic DNA foci were found in close proximity to mitochondria and co-localized with TFAM, a crucial component of mtDNA nucleoids, but not with the nuclear envelope protein lamin B [135]. These structures were absent in rho cells depleted of mtDNA. Therefore, mitochondrial, not nuclear DNA, signals IFN production in these cancer cells [135]. As a proof of concept, IFN production was not achieved in rho cells upon RT and the inhibition of autophagy was unable to restore it [135]. Furthermore, experiments of overexpression and knock-down for the two BCL2 proteins BAK and BAX in breast cancer cell lines showed that mtDNA release and inflammation were mediated by OMM permeabilization and that autophagy did not attenuate the IFN response [135] (Figure 3).

Along the same line of evidence are findings by Giampazolias et al. [131] who showed that, under caspase-deficient conditions, OMM permeabilization exerts potent anti-tumor effects mediated by mtDNA-STING signaling and triggers the production of IFN and NF-κB activation [131]. Additional studies have also reported a boosting of anti-tumor responses via pharmacological inhibition of caspases in combination with cytotoxic treatments [138,139]. More recently, RT induced complete tumor regression via CD8 T cell-mediated IFN response and a protection in the following 60 days in caspase 9 deficient mice [140].

Taken as a whole, these findings point towards a relevant and synergistic role of caspase and autophagy activity during apoptosis through the repression of mtDNA-mediated IFN production, a powerful driver of ICD. These results are very promising as the possibility of eliminating dying cells in situ together with their conversion into elements driving anti-tumor immunity may be not too far from being achieved.

## 6. Conclusions

The displacement of mitochondrial components, including mtDNA, into extracellular compartments has gained considerable attention for its involvement in the modulation of innate immune responses. Due to the bacterial origin of this organelle, mtDNA possesses features of bacterial DNA, such as CpG islands, that are recognized as DAMPs by the innate immune system. However, the mechanisms that regulate the packaging and unloading of mtDNA and other mitochondrial components are still unclear. In physiological conditions, mtDNA is embedded in the mitochondrial matrix in the form of nucleoid structures. Upon cell death-triggering stressors and mPTP opening, mtDNA can be released into the cytoplasm and ignite inflammation via NLRP3, TLR9, and STING pathways. The characterization of the routes through which mitochondrial components may be disposed is highly sought after as these pathways may unveil molecular targets amenable for interventions. In this regard, crosstalk between autophagy and apoptosis has emerged as a converging point for the regulation of cell death and survival. Indeed, this crosstalk represents, under certain circumstances, a relevant molecular node switching immunologically silent into ICD.

## Data Availability

No new data were created or analyzed in this study.
